# A systematic review and meta-analysis of individual patient data on the impact of the BIM deletion polymorphism on treatment outcomes in epidermal growth factor receptor mutant lung cancer

**DOI:** 10.18632/oncotarget.17102

**Published:** 2017-04-13

**Authors:** Sheila X. Soh, Fahad J. Siddiqui, John C. Allen, Go Woon Kim, Jae Cheol Lee, Yasushi Yatabe, Manabu Soda, Hiroyuki Mano, Ross A. Soo, Tan-Min Chin, Hiromichi Ebi, Seiji Yano, Keitaro Matsuo, Xiaomin Niu, Shun Lu, Kazutoshi Isobe, Jih-Hsiang Lee, James C. Yang, Mingchuan Zhao, Caicun Zhou, June-Koo Lee, Se-Hoon Lee, Ji Yun Lee, Myung-Ju Ahn, Tira J. Tan, Daniel S. Tan, Eng-Huat Tan, S. Tiong Ong, Wan-Teck Lim

**Affiliations:** ^1^ Cancer and Stem Cell Biology Program, Duke-NUS Medical School, Singapore; ^2^ Centre for Quantitative Medicine, Duke-NUS Medical School, Singapore; ^3^ Centre for Global Child Health, Sick Kids Hospital, Toronto, Canada; ^4^ Department of Pulmonary and Critical Care Medicine, Asan Medical Center, University of Ulsan, Seoul, Republic of Korea; ^5^ Department of Oncology, Asan Medical Center, University of Ulsan, Seoul, Republic of Korea; ^6^ Department of Pathology and Molecular Diagnostics, Aichi Cancer Center, Nagoya, Japan; ^7^ Department of Cellular Signaling, Graduate School of Medicine, University of Tokyo, Tokyo, Japan; ^8^ Department of Haematology-Oncology, National University Cancer Institute, Singapore; ^9^ Cancer Science Institute, National University of Singapore, Singapore; ^10^ Division of Medical Oncology, Cancer Research Institute, Kanazawa University, Kanazawa, Japan; ^11^ Division of Molecular and Clinical Epidemiology, Aichi Cancer Center Research Institute, Nagoya, Japan; ^12^ Shanghai Lung Cancer Center, Shanghai Chest Hospital, Shanghai Jiao Tong University, Shanghai, China; ^13^ Department of Respiratory Medicine, Toho University Omori Medical Center, Tokyo, Japan; ^14^ Department of Medical Research, National Taiwan University Hospital, Taipei, Taiwan; ^15^ Department of Oncology, Graduate Institute of Oncology and Cancer Research Centre, National Taiwan University Hospital, Taipei, Taiwan; ^16^ Department of Medical Oncology, Shanghai Pulmonary Hospital, Tongji University School of Medicine, Shanghai, China; ^17^ Department of Internal Medicine, Seoul National University Hospital, Seoul, Republic of Korea; ^18^ Division of Hematology and Oncology, Department of Medicine, Samsung Medical Center, Sungkyunkwan University School of Medicine, Seoul, Republic of Korea; ^19^ Division of Medical Oncology, National Cancer Centre, Singapore; ^20^ Department of Haematology, Singapore General Hospital, Singapore; ^21^ Department of Medicine, Duke University Medical Center, Durham, North Carolina, USA

**Keywords:** *BIM*, polymorphism, tyrosine kinase inhibitor, lung cancer, drug resistance

## Abstract

**Background:**

A germline deletion in the *BIM* (*BCL2L11*) gene has been shown to impair the apoptotic response to tyrosine kinase inhibitors (TKIs) *in vitro* but its association with poor outcomes in TKI-treated non-small cell lung cancer (NSCLC) patients remains unclear. We conducted a systematic review and meta-analysis on both aggregate and individual patient data to address this issue.

**Results:**

In an aggregate data meta-analysis (*n* = 1429), the *BIM* deletion was associated with inferior PFS (HR = 1.51, 95%CI = 1.06–2.13, *P* = 0.02). Using individual patient data (*n* = 1200), we found a significant interaction between the deletion and ethnicity. Amongst non-Koreans, the deletion was an independent predictor of shorter PFS (Chinese: HR = 1.607, 95%CI = 1.251–2.065, *P* = 0.0002; Japanese: HR = 2.636, 95%CI = 1.603–4.335, *P* = 0.0001), and OS (HR = 1.457, 95% CI = 1.063–1.997, *P* = 0.019). In Kaplan-Meier analyses, the *BIM* deletion was associated with shorter survival in non-Koreans (PFS: 8.0 months v 11.1 months, *P* < 0.0005; OS: 25.7 v 30.0 months, *P* = 0.042). In Koreans, the *BIM* deletion was not predictive of PFS or OS.

**Materials and Methods:**

10 published and 3 unpublished studies that reported survival outcomes in NSCLC patients stratified according to *BIM* deletion were identified from PubMed and Embase. Summary risk estimates were calculated from aggregate patient data using a random-effects model. For individual patient data, Kaplan-Meier analyses were supported by multivariate Cox regression to estimate hazard ratios (HRs) for PFS and OS.

**Conclusions:**

In selected populations, the *BIM* deletion is a significant predictor of shorter PFS and OS on EGFR-TKIs. Further studies to determine its effect on response to other *BIM*-dependent therapeutic agents are needed, so that alternative treatment strategies may be devised.

## INTRODUCTION

While sensitizing mutations in the *EGFR* gene predict very high response rates among patients with NSCLC [1–3], up to 30% of such patients fail to experience optimum responses [4, 5]. The identification of factors which predict patient response is important for both clinical and economic reasons [6–8], and is especially relevant for East Asian populations where the incidence of *EGFR*-mutated NSCLC is at least 4-fold higher than among non-East Asian populations [9].

Several mechanisms to explain the heterogeneity of TKI responses have been previously described [10–13]. In particular, impaired expression/function of BIM (BCL2L11), resulting in failure of TKI-induced apoptosis has been associated with TKI resistance in preclinical models [14–18]. BIM is a pro-apoptotic protein that is upregulated upon TKI treatment and binds to pro-survival BCL-2 family members via its BH3 domain, thereby initiating programmed cell death [19]. Accordingly, BIM expression has been found to represent a key node in determining responses to cancer drug therapy in general [20]. Of note, recent data from the European Tarceva (EURTAC) trial demonstrated poorer outcomes in *EGFR*-mutant NSCLC patients with low *BIM* expression, further supporting the importance of intact apoptotic machinery in such populations [21]. Together, these studies highlight BIM function as a critical factor in determining patient responses to cancer therapies.

Recently, a 2.9kb intronic deletion in the *BIM* gene that biases splicing towards non-apoptotic isoforms lacking the BH3 domain was discovered [22]. This deletion polymorphism appeared to occur at a frequency of 12.3% in East Asians but was absent in Caucasian populations [22]. When introduced into NSCLC cell lines, the deletion resulted in an increase in the production of non-apoptotic isoforms at the expense of apoptotic isoforms, and relative resistance to EGFR-TKIs [22]. Importantly, it has also been shown that resistance conferred by the deletion could be overcome *in vitro* with HDAC inhibitors [23], and BH3 mimetics [22]. It was therefore hypothesised that the *BIM* deletion would have an impact on the sensitivity of EGFR-addicted tumors to EGFR-TKIs, and indeed the polymorphism was associated with shorter progression-free survival (PFS) in a retrospective EGFR-TKI-treated NSCLC cohort from Singapore and Japan [22].

Since then, other centers across East Asia, including China and Taiwan, have investigated the association between the *BIM* polymorphism and treatment outcome in both prospective and retrospective TKI-treated NSCLC patient cohorts, with conflicting results [24–30]. Notably, the two studies that reported a lack of association originated from South Korea, raising the question if the effect of the deletion may be modulated by ethnicity. Furthermore, although the *BIM* deletion was initially thought to be exclusive to East Asian populations, it has been recently reported in a Columbian population [31]. Hence, an understanding of the true effect of the deletion on TKI treatment outcomes in different ethnic populations is important for predicting response and guiding treatment strategies in East Asian populations, and potentially beyond East Asian populations.

A number of aggregate data meta-analyses that have been published have suggested that the *BIM* polymorphism is indeed associated with shorter PFS on EGFR-TKIs [32–35]. However, these meta-analyses were limited by the use of summary statistics from published studies. The alternative approach, based on individual patient data, offers several clinical and statistical advantages [36]. In this collaborative study, we used individual patient data from published and unpublished studies to perform a meta-analysis on the effect of the *BIM* polymorphism on PFS and OS in EGFR-TKI-treated NSCLC patients as the primary aim.

## RESULTS

### Search strategy

**Figure 1:**
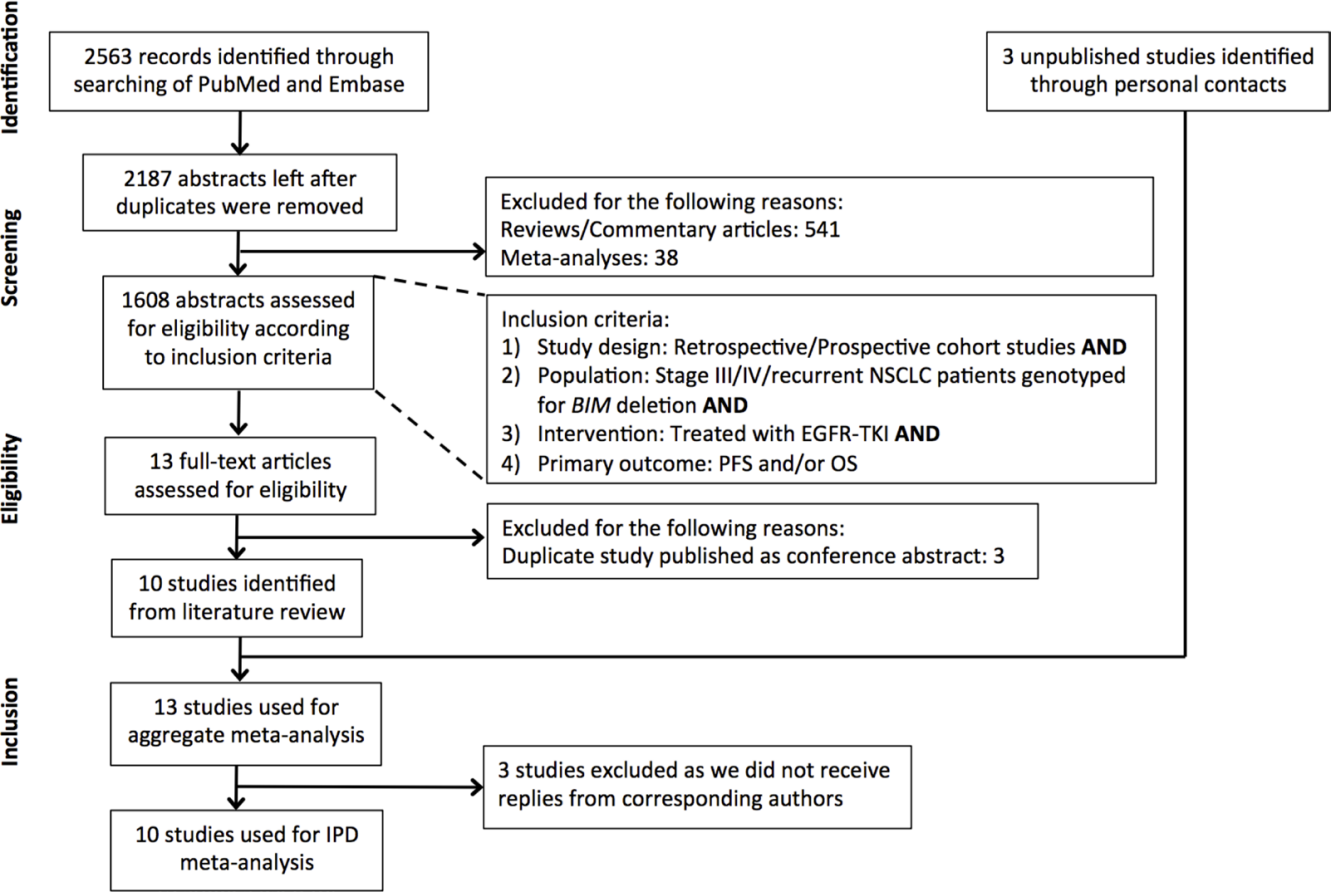
Flowchart of study identification, inclusion and exclusion Studies identified from a PubMed and Embase search were filtered according to the exclusion and inclusion criteria listed in the Figure. In total, 10 studies were available for individual patient data (IPD) analysis.

Figure 1 illustrates the search strategy used to identify studies that investigated the association of the *BIM* deletion with responses among individuals with *EGFR*-mutant NSCLC. A total of 2187 unique articles were identified from PubMed and Embase after removing duplicate abstracts. 13 articles were selected for full-text review after inclusion and exclusion criteria were applied. 10 studies were eventually identified and the corresponding authors were contacted. We received individual patient data for 7 of these studies [22, 25–28, 30, 37]. Authors of 3 studies did not reply (*n* = 502) [24, 29, 31]. Published data was supplemented with 3 additional unpublished datasets from China, Japan and Singapore (SL, SY, WTL).

Table 1 summarizes the characteristics of the 13 studies we identified. In total, individual patient data from 1324 patients was received. We excluded patients if they were not treated with TKIs, the treatment period was < 2 weeks, or *BIM* deletion status was unknown. Duplicate patients were also excluded (Supplementary Table 1). Eventually, data from 1200 NSCLC patients (representing 90.6% of the total number of patients) was used for a pooled individual patient data meta-analysis, to determine PFS and OS on EGFR-TKIs in the whole cohort.

**Table 1:** Characteristics of studies identified for the meta-analysis

Author	Year	Country	Sample size	Patients with *BIM* deletion (%)	Line of TKI treatment
Ng KP et al.	2012	Singapore, Japan	141	26 (18.4%)	First-line or later
Lee JK et al.	2013	South Korea	197	21 (10.7%)	First-line or later
Zheng L et al.*	2013	China	123	21 (17.1%)	Second-line or later
Isobe K et al.	2014	Japan	70	13 (18.6%)	First-line or later
Lee JH et al.†	2014	Taiwan	153	27 (17.6%)	First-line only
Zhao MC et al.	2014	China	166	16 (9.6%)	First-line or later
Zhong J et al.*	2014	China	290	45 (15.5%)	First-line or later
Cardona AF et al.*	2014	Colombia	89	14 (15.7%)	Not reported
Kim GW et al.	2015	South Korea	21	4 (19%)	First-line or later
Lee JY et al.	2015	South Korea	205	32 (15.6%)	First-line or later
Yano S et al.	Unpublished	Japan	39	6 (15.4%)	First-line or later
Lu S et al.	Unpublished	China	55	9 (16.4%)	First-line or later
Lim WT et al.	Unpublished	Singapore	178	22 (12.4%)	First-line or later

Legend:

*Authors did not respond to requests for data.

^†^Prospective study.

### Baseline characteristics of patients with and without the BIM deletion

Baseline characteristics of the cohort as a whole, and segregated according to *BIM* deletion status, are detailed in Supplementary Table 2. Patients with or without the *BIM* deletion did not differ significantly (defined as *p* > 0.05) in terms of the characteristics examined, including established prognostic factors for *EGFR*-mutant NSCLC [38]. All the studies used for this pooled analysis used data from stage III/IV NSCLC patients on EGFR-TKI monotherapy. The majority of patients (96.4%) were treated with standard doses of either gefitinib or erlotinib. Although the TKI used differed according to each country's practices, similar outcomes have been reported in earlier studies that compared gefitinib to erlotinib [39, 40].

### Progression-free survival on EGFR-TKIs in patients with and without the BIM deletion

**Figure 2:**
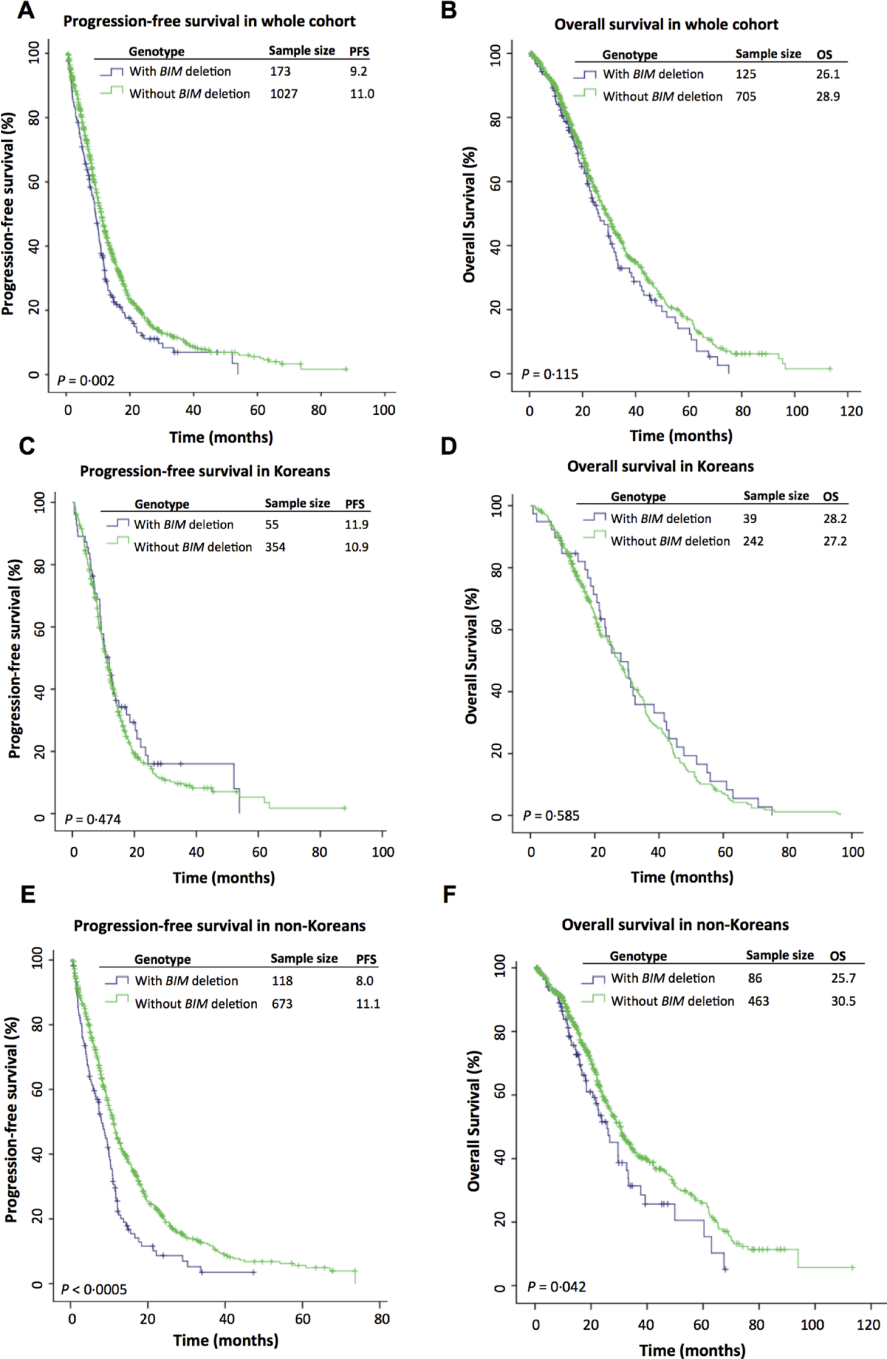
Kaplan-Meier curves comparing progression-free survival (PFS) and overall survival (OS) on EGFR-TKIs between patients with and without the *BIM* deletion in the (**A**, **B**) whole cohort, (**C**, **D**) Korean cohort and (**E**, **F**) non-Korean cohort.

We first performed a Kaplan-Meier analysis on the pooled individual patient dataset (*n* = 1200) to determine the PFS on EGFR-TKIs of patients segregated according to previously reported prognostic factors, in particular, the *BIM* deletion. We found that patients with the *BIM* deletion had a significantly shorter median PFS of 9.2 months compared to 11.0 months in those without the deletion (*P* = 0.002) (Figure 2A). As expected, log-rank *p* values for Kaplan-Meier analyses were also significant for well-validated predictors of survival such as histology (*P* = 0.001), *EGFR* phenotype (*P* < 0.0005), gender (*P* < 0.0005), smoking history (*P* = 0.024), and stage (*P* < 0.005). In addition, country (*P* < 0.0005), center (*P* < 0.0005), ethnicity (*P* < 0.0005) also emerged as significant predictors of PFS (data not shown).

Considering that ethnicity subsumes center and country, we focussed on investigating the effect of the *BIM* deletion stratified by ethnicity (Supplementary Figure 1), and found that Korean patients with and without the deletion—contrary to the other ethnicities—had similar PFS (11.9 *v* 10.9 months; *P* = 0.474) (Figure 2C). In contrast, an analysis of non-Korean patients showed a significant difference in PFS in patients with and without the *BIM* deletion (8.*0* *v* 11.1 months; *P* < 0.0005) (Figure 2E).

To support our findings from the Kaplan-Meier analysis, we used Cox regression analysis to identify significant factors associated with shorter PFS in patients on EGFR-TKI treatment. Here, the *BIM* deletion emerged as a significant risk factor for reduced PFS in both the univariate (HR = 1.323, 95% CI = 1.108–1.581, *P* = 0.002) and stepwise multivariate Cox regression analyses (HR = 1.406, 95% CI = 1.174–1.684, *P* < 0.0005) (Table 2). To explore the extent to which ethnicity moderated the effect of the *BIM* deletion on PFS, an interaction term (*BIM* deletion x ethnicity) was incorporated into the Cox regression model (Table 3). This analysis yielded significant main effects for the *BIM* deletion (*P* = 0.0002) and ethnicity (*P* = 0.0020), as well as a significant interaction between *BIM* deletion and ethnicity (*P* = 0.0023). The effect of the *BIM* deletion differed according to ethnicity, as it was a significant risk factor for reduced PFS in Chinese (HR = 1.607, 95% CI = 1.251–2.065, *P* = 0.0002) and Japanese (HR = 2.636, 95% CI = 1.603–4.335, *P* = 0.0001), but not Korean patients (HR = 0.919, 95% CI = 0.668–1.264, *P* = 0.603) (Table 3).

**Table 2:** Results of univariate and multivariate analyses for predictors of PFS, whole cohort

	Univariate Analysis			Multivariate Analysis		
	HR (95% CI)		*p* value	HR (95% CI)		*p* value
**Age at diagnosis**	0.992	(0.987−0.998)	0.012	0.989	(0.983−0.995)	< 0.0005
**Gender (Ref = Female)**						
Male	1.359	(1.190−1.550)	< 0.0005	1.355	(1.186−1.547)	< 0.0005
**Ethnicity (Ref = Others)**						
Chinese	1.048	(0.72−1.525)	0.806	1.047	(0.717−1.529)	0.810
Japanese	0.678	(0.448−1.026)	0.066	0.796	(0.525−1.209)	0.285
Korean	0.952	(0.651−1.392)	0.800	1.162	(0.79−1.708)	0.446
**Smoking history (Ref = Never smoker)**						
Ever smoker	1.228	(1.059−1.425)	0.007	−		−
**Stage (Ref = Relapse)**						
IIIA/B	1.583	(1.201−2.087)	0.001	1.523	(1.147−2.022)	0.004
IV	1.412	(1.175−1.699)	< 0.0005	1.455	(1.201−1.762)	< 0.0005
**Histology (Ref = Adenocarcinoma)**						
Non-adenocarcinoma	1.590	(1.203−2.101)	0.001	1.832	(1.381−2.433)	< 0.0005
**EGFR phenotype (Ref = Sensitizing)**						
Resistant	2.551	(1.972−3.300)	< 0.0005	2.705	(2.067−3.542)	< 0.0005
**Line of treatment (Ref = Later)**						
First-line	1.152	(1.010−1.313)	0.035	−		−
**ECOG status (Ref = 0 or 1)**						
≥ 2	1.284	(0.997−1.653)	0.053	1.408	(0.898−2.209)	0.136
***BIM* deletion (Ref = Absent)**						
Present	1.323	(1.108−1.581)	0.002	1.406	(1.174−1.684)	< 0.0005

**Table 3:** Significant predictors of PFS on EGFR-TKIs, whole cohort, using a *BIM* deletion x ethnicity interaction term

	Multivariate Analysis	
	HR (95% CI)	*p* value
**Age at diagnosis**	0.989 (0.983–0.995)	0.0002
**Gender (Ref = Female)**		
Male	1.325 (1.160–1.513)	< 0.0001
**Stage (Ref = Relapse)**		
IIIA/B	1.515 (1.141–2.012)	0.0041
IV	1.408 (1.163–1.705)	0.0005
**Histology (Ref = Adenocarcinoma)**		
Non-adenocarcinoma	1.858 (1.399–2.468)	< 0.0001
**EGFR phenotype (Ref = Sensitising)**		
Resistant	2.739 (2.093–3.585)	< 0.0001
**ECOG status (Ref = 0 or 1)**		
≥ 2	1.428 (1.106–1.844)	0.0063
**BIM deletion x ethnicity (Ref = Absent)**		
Chinese	1.607 (1.251–2.065)	0.0002
Japanese	2.636 (1.603–4.335)	0.0001
Korean	0.919 (0.668–1.264)	0.6030
Others	2.115 (0.804–5.564)	0.1290

### Baseline characteristics of patients of Korean and non-Korean ethnicity

To exclude differences in patient population as a reason for the discordant results, we compared the baseline characteristics of patients of Korean and non-Korean ethnicity. We found that the Korean and non-Korean cohorts differed in terms of ECOG status, histology, *EGFR* mutation, TKI used and line of treatment (Supplementary Table 3). We then performed subgroup Kaplan-Meier analyses using only data from patients who had 1) ECOG status 0 or 1, 2) adenocarcinoma histology, 3) TKI-sensitizing *EGFR* mutations, 4) gefitinib and/or erlotinib treatment or 5) first-line treatment with TKIs (Supplementary Table 4). In these subgroup analyses, the *BIM* deletion remained a significant predictor of shorter PFS in only the non-Korean patients, and not the Korean patients. Hence, it is unlikely that these differences in baseline characteristics contribute to the discordant effects of the deletion on PFS in Korean and non-Korean patients.

### Overall survival on EGFR-TKIs in patients with and without the BIM deletion

In earlier studies, there had been conflicting results as to whether the *BIM* deletion predicted poorer overall survival [27, 28, 30]. Here, for the cohort as a whole, there was no significant difference in OS in patients with the *BIM* deletion compared to those without for whom overall survival data was available (*n* = 830) (26.1 *v* 28.9 months, *P* = 0.115) (Figure 2B). However, when stratified by Korean ethnicity, there was a significant difference in OS amongst non-Koreans with and without the *BIM* deletion (25.*7* *v* 30.5 months, *P* = 0.042) (Figure 2F) but not amongst Koreans (28.2 *v* 27.2 months, *P* = 0.585) (Figure 2D). In a multivariate Cox regression analysis, the *BIM* deletion emerged as a significant predictor of shorter OS (HR = 1.457, 95% CI = 1.063–1.997, *P* = 0.019) in non-Korean patients (*n* = 549) (Table 4).

**Table 4:** Results of univariate and multivariate analyses for predictors of OS, non−Koreans

	Univariate Analysis		Multivariate Analysis		
	HR (95% CI)		*p* value	HR (95% CI)	*p* value
**Age at diagnosis**	1.009	(0.999−1.019)	0.095	−	−
**Gender (Ref = Female)**					
Male	1.453	(1.149−1.838)	0.002	1.490 (1.175−1.890)	0.001
**Ethnicity (Ref = Others)**					
Chinese	0.806	(0.438−1.484)	0.489	−	−
Japanese	0.628	(0.329−1.198)	0.158	−	−
**Smoking history (Ref = Never smoker)**					
Ever smoker	1.215	(0.940−1.570)	0.136	−	−
**Stage (Ref = Relapse)**					
IIIA/B	1.286	(0.754−2.193)	0.356	−	−
IV	1.285	(0.892−1.851)	0.178	−	−
**Histology (Ref = Adenocarcinoma)**					
Non-adenocarcinoma	2.160	(1.408−3.311)	< 0.0005	2.551 (1.653−3.937)	< 0.0005
**EGFR phenotype (Ref = Sensitising)**					
Resistant	1.881	(1.378−2.566)	< 0.0005	1.939 (1.413−2.661)	< 0.0005
**Line of treatment (Ref = Later)**					
First-line	1.453	(1.081−1.952)	0.013	1.409 (1.041−1.907)	0.026
**ECOG status (Ref = 0 or 1)**					
≥ 2	2.370	(31.538−3.650)	< 0.0005	2.744 (2.464−3.056)	0.004
***BIM* deletion (Ref = Absent)**					
Present	1.378	(1.010−1.881)	0.043	1.457 (1.063−1.997)	0.019

### Aggregate data meta-analysis

**Figure 3:**
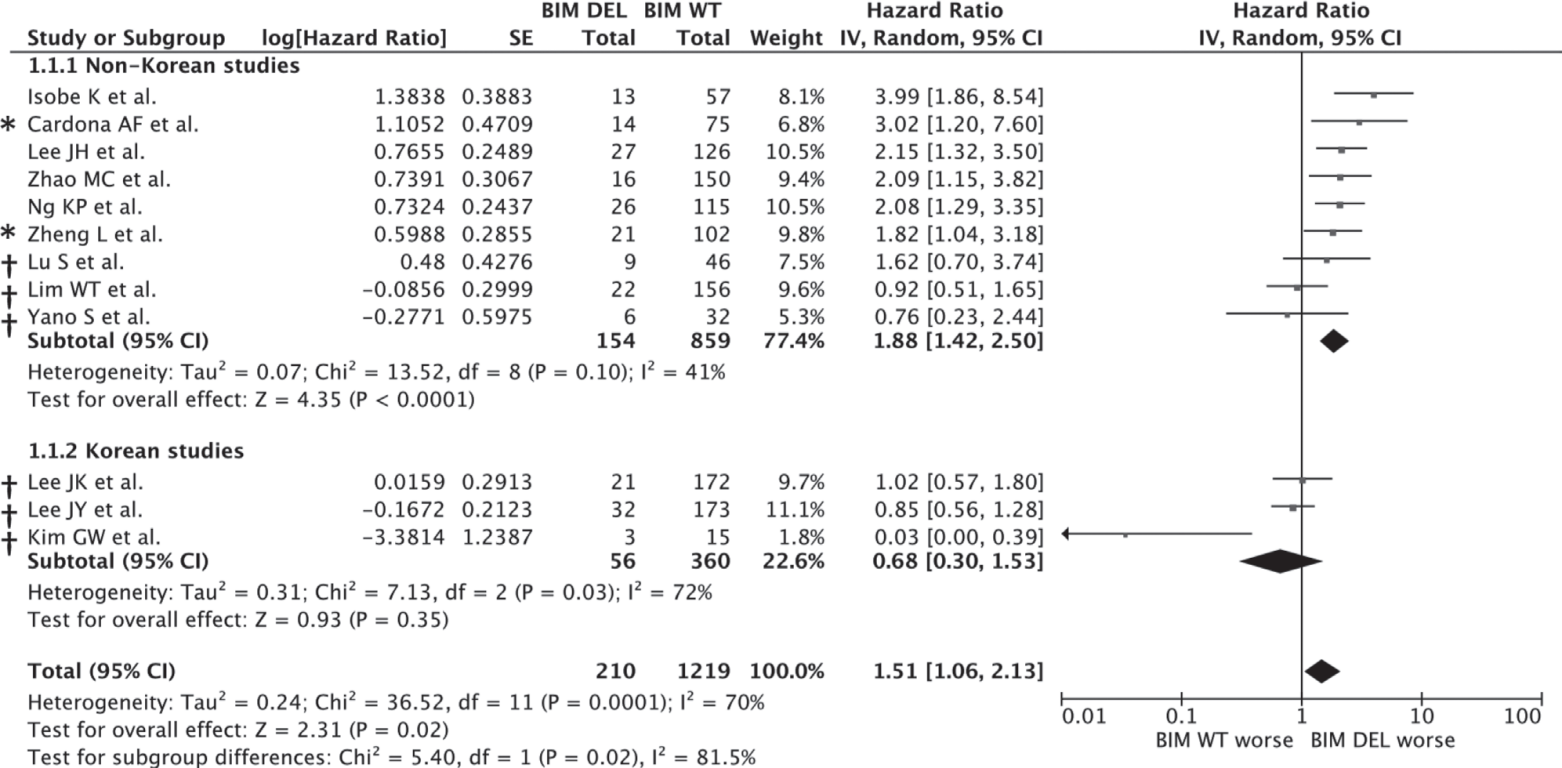
Cumulative meta-analysis of published and unpublished data of the association between the *BIM* deletion polymorphism and progression-free survival in EGFR-TKI-treated NSCLC patients Estimates were adjusted for age, sex, smoking history, stage, ECOG status, histology, *EGFR* mutation and line of treatment. Zhong J *et al*. was omitted from this figure as both summary statistics and individual patient data (IPD) were not available. * indicates IPD was unavailable, † indicates summary HRs were calculated by the authors of this paper according to the methods described in the Methods section.

In view of potential study heterogeneity, and in order to include as many studies as possible (including the authors who did not reply to requests for IPD), we performed a meta-analysis using a random effects model based on aggregate patient data (*n* = 1429). Consistent with results from the pooled IPD analysis, the presence of the *BIM* deletion was associated with a significant overall increase in the risk of progression compared to individuals without the *BIM* deletion (HR = 1.51, 95% CI = 1.06–2.13, *P* = 0.02) in an aggregate data meta-analysis (Figure 3).

However, because there was significant heterogeneity within the combined group (I^2^ = 70%, *P* = 0.0001) (Figure 3), and because the 2 studies to date that reported a lack of association between the *BIM* deletion and treatment outcome originated from Korean centers, we tested the possibility that the heterogeneity arose from differences in non-Korean *v* Korean groups. Consistent with this notion, the non-Korean groups were found to be homogenous when analyzed separately (I^2^ = 41%, *P* = 0.10) (Figure 3). Importantly, and consistent with the results from the individual patient data-based analysis, we also found that the summary HR for risk of shorter PFS was significant in patients with the deletion in the non-Korean studies (HR = 1.88, 95% CI: 1.42–2.50, *P* < 0.0001), whereas the deletion was not a significant risk factor for shorter PFS in Koreans (HR = 0.68, 95% CI = 0.30–1.53, *P* = 0.35) (Figure 3).

## DISCUSSION

In the current work, we have used both pooled individual patient data as well as aggregate patient data to show that the germline *BIM* deletion, like TKI-resistant *EGFR* mutations, predicts inferior outcomes in selected populations. Our findings indicate that Chinese and Japanese patients with *EGFR*-mutant NSCLC who carry the *BIM* deletion polymorphism have an inferior progression-free survival compared to those who do not (Chinese: HR = 1.607, 95% CI = 1.251–2.065, *P* = 0.0002; Japanese: HR = 2.636, 95% CI = 1.603–4.335, *P* = 0.0001). Importantly, this remains the only germline variant known to reproducibly affect clinical outcomes in *EGFR*-mutant NSCLC across multiple studies [41]. In contrast, several germline polymorphic variants in ABC transporter genes which may influence EGFR-TKI pharmacokinetics have not carried the same effect [42].

To assess the impact of the deletion in the Korean population, we combined individual patient data from two Korean studies [25, 30], as well as an additional cohort (*n* = 18) from another Korean center [37]. The lack of effect of the deletion in the Korean studies could account for some of the statistical heterogeneity seen in our random-effects model (Figure 3), and the use of individual patient data has been invaluable in allowing a more accurate quantification of the effect of the *BIM* deletion on PFS according to ethnicity (Table 3).

Several possibilities may account for the discordant effects of the deletion between Korean and non-Korean populations. Firstly, the discordant effects seen in different ethnic populations may be a result of differences in treatment protocols between countries, rather than ethnicity (Korean *v* non-Korean) per se. However, this is unlikely, as to our knowledge, EGFR-TKI treatment protocols of Korean and non-Korean centers are relatively similar. Although the Korean and non-Korean populations differed in terms of other baseline characteristics other than ethnicity (Supplementary Table 3), subgroup analysis using only patients who had the same ECOG status, histology, sensitizing *EGFR* mutations, TKI used and line of treatment showed a significantly shorter PFS in non-Korean patients with the *BIM* deletion, but not Korean patients.

If the discordant effect of the deletion truly arises from differences in genetic makeup between non-Koreans and Koreans, a possible explanation is the existence of other genetic factors in linkage disequilibrium with the *BIM* deletion that has occurred only in Korean populations through the process of genetic drift and divergence from the other East Asian populations. Such factors may modulate the effect of the deletion on TKI responses, and may also include Korean-specific single nucleotide polymorphisms (SNPs) in *BIM* itself that counter the effects of the *BIM* deletion. Of relevance to this discussion, functional polymorphisms in *BIM* besides the deletion polymorphism have been reported in other populations [43, 44]. It is also possible that functional *BIM* SNPs may exist at varying frequencies between different East Asian populations, as has been reported for functional SNPs affecting genes other than *BIM* [45].

Another factor we could not correct for despite the availability of individual patient data is the frequency of radiological imaging used to determine tumor response or progression. In this instance, length time bias may be introduced by differences in the frequency of imaging, and can only be properly addressed through large prospective studies employing imaging at regular and pre-defined intervals [46].

In addition to kinase inhibitors, the effect of the *BIM* deletion could potentially extend to chemotherapeutics. This possibility is consistent with the superior overall survival we observed in non-Korean patients without the deletion (Figure 2F and Table 4) since patients would very likely have received chemotherapy during the course of their disease. Importantly, this clinical finding is supported by preclinical data demonstrating that sensitivity to taxanes and vinca alkaloids, which are commonly used in the treatment of NSCLC patients, depends on BIM expression [47, 48]. It would therefore be interesting in future studies to examine the effect of the *BIM* deletion on PFS on chemotherapy. If the *BIM* deletion did indeed influence treatment outcomes beyond TKIs, genotyping for the *BIM* deletion may be a recommendation for any therapeutic strategy that depends on BIM function for efficacy. Furthermore, a recent Japanese study has highlighted a correlation between the *BIM* deletion and relapse-free survival in patients with early-stage resected lung cancer [49], suggesting possible implications of the *BIM* deletion on adjuvant therapy in early-stage lung cancer, where vinorelbine is widely used.

Knowledge about EGFR-TKI resistance has evolved since the discovery of the *BIM* deletion and the understanding that low frequency *MET* amplification (< 5%) [50] and *de novo* T790M mutations (1–2%) [51] contribute to a small percentage of intrinsic resistance. These areas are not addressed in this meta-analysis because presence of these resistance mechanisms in individual patients is not routinely assessed in clinical practice as yet. Nevertheless, apoptosis remains the final common pathway by which inhibitors of MET or EGFR work, hence assessment of the *BIM* deletion status in populations where it is common remains relevant. Indeed, ongoing clinical trials are currently testing the hypothesis that combination therapy using EGFR TKIs and HDAC inhibitors will restore TKI sensitivity to patients with the *BIM* deletion polymorphism [23].

There is also preliminary evidence suggesting that the impact of our results may extend beyond East Asian populations. The *BIM* deletion has been reported to be present in a South American population at a frequency of 15.3% [31]. Accordingly, the effect of the *BIM* deletion among the South American population should also be examined, particularly since the *EGFR* mutation rate and therefore proportion of EGFR-TKI-responsive lung cancers in South American populations is higher than reported in European studies [52].

In conclusion, our analysis confirms that patients with *EGFR*-mutant NSCLC harboring the *BIM* deletion are at higher risk for reduced progression-free survival compared to those without the deletion. In addition, the association is strongest among Chinese and Japanese individuals for whom the *BIM* deletion also predicts a reduction in overall survival. Accordingly, testing for the presence of the *BIM* deletion among patients of Chinese or Japanese ancestry with *EGFR*-mutant NSCLC may have prognostic utility. Finally, ongoing trials (clinicaltrials.gov/ct2/show/NCT02151721) are testing the hypothesis that combination therapy with HDAC inhibitors and EGFR-TKIs will overcome inferior outcomes conferred by the *BIM* deletion. If positive, such studies will support the use of the *BIM* deletion as a biomarker for combination therapy.

## MATERIALS AND METHODS

### Literature search and data extraction

A bibliographic search with no language restrictions was made on PubMed and Embase to identify eligible studies. As the first paper to describe the *BIM* polymorphism was published in 2012, the search was restricted to papers published in or after 2012, up to September 21, 2015. The search strategy was developed by FJS, and was run using terms related to lung cancer (“Lung Cancer” or “Carcinoma, Non-Small-Cell Lung” or “Lung Neoplasms” or “Lung Adenocarcinoma” or “EGFR lung cancer” or “EGFR mutant lung cancer”), tyrosine kinase inhibitors (“tyrosine kinase inhibitor or “kinase inhibitor” or “TKI” or “Iressa” or “Tarceva” or “gefitinib” or “erlotinib” and *BIM* polymorphism (“BIM” or “BCL2L11” OR “BCL-2-like protein” or “*BIM* polymorphism” or “BCL2L11 polymorphism”).

Two authors (SXS and WTL) independently screened the title and abstract of retrieved articles. Articles were included if they met the following criteria: 1) study design: prospective or retrospective cohort studies, 2) population: patients with advanced stage (IIIA/B/IV) or postoperative relapsed NSCLC and known *BIM* deletion status, 3) intervention: EGFR-TKI monotherapy and 4) primary outcome: PFS and/or OS. Meta-analyses, case studies, reviews, commentary articles and replies were excluded from the analysis. Studies that cleared the title and abstract screening stages were selected for full-text review. Disagreements were resolved by discussion between the two reviewers.

### Quality and bias assessment

We used the Cochrane Risk of Bias Tool to assess the quality of the studies included (Supplementary Table 5). The quality of the study was regarded as high if all aspects were assessed favorably.

To minimize publication bias, and as suggested by the Cochrane Handbook of Systemic Reviews of Interventions [53], we have taken the following measures. First, we performed a comprehensive search on PubMed and Embase with no language restrictions, and used multiple synonyms of search terms. Secondly, and because the research topic here is a highly specialized one with only a small community of researchers in East Asia who are known to each other, we were able to obtain data from 3 unpublished studies for inclusion in the meta-analysis.

### Individual patient data collection

Following ethics approval by the SingHealth Institutional Review Board (IRB), invitations to participate in the meta-analysis were sent to all investigators of the primary studies identified through the literature search. Corresponding authors were responsible for their individual IRB approvals. Each author was requested to submit anonymized individual patient data in a Microsoft Excel template which included the following fields: age at diagnosis, gender and ethnicity of the patient, smoking history, tumor stage at diagnosis, histology, *BIM* deletion status, *EGFR* mutation, EGFR-TKI used, line of treatment, ECOG status, date TKI was started/stopped, reason TKI was stopped, PFS, date of diagnosis, date of last follow-up, date of death, and OS. Any inconsistencies that were identified were resolved through email correspondence with the respective corresponding authors.

### Definition of outcomes

The primary outcome, PFS, was defined as the length of time from the date TKI was started to either the date TKI was stopped or date of tumor progression, depending on availability of data. PFS data was considered ‘mature’ if treatment was stopped because of progression, and ‘censored’ if TKI treatment was stopped due to side-effects or was ongoing. The secondary outcome, OS, was defined as the length of time from the date first-line therapy (chemotherapy or TKI) was initiated for advanced disease until death from any cause. OS data was censored if the patient was still alive on the date of last follow-up.

### Statistical analysis

The statistical analyses were carried out using SAS Version 9.3 (SAS^®^ Cary, NC, USA), SPSS Version 20 (IBM, NY, USA) and Review Manager (RevMan) Version 5.3 (Copenhagen: The Nordic Cochrane Centre, The Cochrane Collaboration, 2014). Differences in baseline characteristics of patients with and without the deletion, or with and without Korean ethnicity, were assessed using two-sample *t*-tests, Fisher's exact test and Pearson Chi-Square test where appropriate. Kaplan-Meier curves for factors reported in the literature to have an impact on survival were compared using the log-rank test. In addition, to evaluate potential differences possibly arising from including patients from different centers, survival was assessed among countries, centers, and ethnicities. As we did not find evidence that the effect of *BIM* deletion on PFS was modulated by the type of TKI-sensitizing *EGFR* mutation in a Cox regression model incorporating type of *EGFR* mutation, *BIM* deletion and an interaction term *EGFR* mutation x *BIM* deletion (*P* = 0.906), for our analyses, we grouped the patients into those with sensitizing mutations, and those without. *EGFR* phenotype was defined as sensitizing if mutations in exon 18, 19 and 21 were present, and resistant if *EGFR* was wildtype or contained non-synonymous mutations in exon 20.

Univariate HRs were calculated for the following factors: age, gender, smoking history, stage of disease, histology, *EGFR* phenotype (sensitizing or resistant), line of treatment, ECOG status and *BIM* genotype. Owing to the exploratory nature of the analysis, significant factors (*P* < 0.2) were entered into a multivariate Cox regression analysis using a forward stepwise selection method and adjusted HRs and 95% CIs were obtained for the association between the *BIM* deletion and PFS.

To make the most of the available evidence in the absence of individual patient data for some of the studies, an aggregate data meta-analysis was also carried out. Adjusted HRs reported in the original papers were used when available. For the remaining studies, adjusted HRs for the *BIM* deletion were calculated using a Cox proportional hazards model that incorporated the following factors if significant (*P* < 0.2): age, gender, smoking history, stage of disease, histology, *EGFR* phenotype (sensitizing or resistant), line of treatment, and ECOG status (Supplementary Tables 6 to 11). In anticipation of heterogeneity in exposure and treatment-related variables, a random-effects model was employed to calculate summary HRs and 95% CIs for PFS.

## SUPPLEMENTARY MATERIALS FIGURES AND TABLES



## Abbreviations

TKI: Tyrosine Kinase Inhibitor
ECOG: Eastern Cooperative Oncology Group
EGFR: Epidermal Growth Factor Receptor
BIM: Bcl-2 Interacting Mediator
NSCLC: Non-Small Cell Lung Cancer
PFS: Progression-free survival
OS: Overall survival
IPD: Individual Patient Data.

## References

[1] Pao W, Miller V, Zakowski M, Doherty J, Politi K, Sarkaria I, Singh B, Heelan R, Rusch V, Fulton L, Mardis E, Kupfer D, Wilson R (2004). EGF receptor gene mutations are common in lung cancers from “never smokers” and are associated with sensitivity of tumors to gefitinib and erlotinib. Proc Natl Acad Sci USA.

[2] Lynch TJ, Bell DW, Sordella R, Gurubhagavatula S, Okimoto RA, Brannigan BW, Harris PL, Haserlat SM, Supko JG, Haluska FG, Louis DN, Christiani DC, Settleman J (2004). Activating mutations in the epidermal growth factor receptor underlying responsiveness of non-small-cell lung cancer to gefitinib. N Engl J Med.

[3] Paez JG, Janne PA, Lee JC, Tracy S, Greulich H, Gabriel S, Herman P, Kaye FJ, Lindeman N, Boggon TJ, Naoki K, Sasaki H, Fujii Y (2004). EGFR mutations in lung cancer: correlation with clinical response to gefitinib therapy. Science.

[4] Mok TS, Wu YL, Thongprasert S, Yang CH, Chu DT, Saijo N, Sunpaweravong P, Han B, Margono B, Ichinose Y, Nishiwaki Y, Ohe Y, Yang JJ (2009). Gefitinib or carboplatin-paclitaxel in pulmonary adenocarcinoma. N Engl J Med.

[5] Rosell R, Carcereny E, Gervais R, Vergnenegre A, Massuti B, Felip E, Palmero R, Garcia-Gomez R, Pallares C, Sanchez JM, Porta R, Cobo M, Garrido P (2012). Erlotinib versus standard chemotherapy as first-line treatment for European patients with advanced EGFR mutation-positive non-small-cell lung cancer (EURTAC): a multicentre, open-label, randomised phase 3 trial. Lancet Oncol.

[6] Gainor JF, Shaw AT (2013). Emerging paradigms in the development of resistance to tyrosine kinase inhibitors in lung cancer. J Clin Oncol.

[7] Soo RA, Anderson BO, Cho BC, Yang CH, Liao M, Lim WT, Goldstraw P, Mok TS, Asian Oncology S (2009). First-line systemic treatment of advanced stage non-small-cell lung cancer in Asia: consensus statement from the Asian Oncology Summit 2009. Lancet Oncol.

[8] de Lima Lopes G, Segel JE, Tan DS, Do YK, Mok T, Finkelstein EA (2012). Cost-effectiveness of epidermal growth factor receptor mutation testing and first-line treatment with gefitinib for patients with advanced adenocarcinoma of the lung. Cancer.

[9] Shigematsu H, Lin L, Takahashi T, Nomura M, Suzuki M, Wistuba II, Fong KM, Lee H, Toyooka S, Shimizu N, Fujisawa T, Feng Z, Roth JA (2005). Clinical and biological features associated with epidermal growth factor receptor gene mutations in lung cancers. J Natl Cancer Inst.

[10] Wu JY, Wu SG, Yang CH, Gow CH, Chang YL, Yu CJ, Shih JY, Yang PC (2008). Lung cancer with epidermal growth factor receptor exon 20 mutations is associated with poor gefitinib treatment response. Clin Cancer Res.

[11] Yasuda H, Kobayashi S, Costa DB (2012). EGFR exon 20 insertion mutations in non-small-cell lung cancer: preclinical data and clinical implications. Lancet Oncol.

[12] Yano S, Wang W, Li Q, Matsumoto K, Sakurama H, Nakamura T, Ogino H, Kakiuchi S, Hanibuchi M, Nishioka Y, Uehara H, Mitsudomi T, Yatabe Y (2008). Hepatocyte growth factor induces gefitinib resistance of lung adenocarcinoma with epidermal growth factor receptor-activating mutations. Cancer Res.

[13] Engelman JA, Zejnullahu K, Mitsudomi T, Song Y, Hyland C, Park JO, Lindeman N, Gale CM, Zhao X, Christensen J, Kosaka T, Holmes AJ, Rogers AM (2007). MET amplification leads to gefitinib resistance in lung cancer by activating ERBB3 signaling. Science.

[14] Kuroda J, Puthalakath H, Cragg MS, Kelly PN, Bouillet P, Huang DC, Kimura S, Ottmann OG, Druker BJ, Villunger A, Roberts AW, Strasser A (2006). Bim and Bad mediate imatinib-induced killing of Bcr/Abl+ leukemic cells, and resistance due to their loss is overcome by a BH3 mimetic. Proc Natl Acad Sci USA.

[15] Cragg MS, Kuroda J, Puthalakath H, Huang DC, Strasser A (2007). Gefitinib-induced killing of NSCLC cell lines expressing mutant EGFR requires BIM and can be enhanced by BH3 mimetics. PLoS Med.

[16] Gong Y, Somwar R, Politi K, Balak M, Chmielecki J, Jiang X, Pao W (2007). Induction of BIM is essential for apoptosis triggered by EGFR kinase inhibitors in mutant EGFR-dependent lung adenocarcinomas. PLoS Med.

[17] Costa DB, Halmos B, Kumar A, Schumer ST, Huberman MS, Boggon TJ, Tenen DG, Kobayashi S (2007). BIM mediates EGFR tyrosine kinase inhibitor-induced apoptosis in lung cancers with oncogenic EGFR mutations. PLoS Med.

[18] Faber AC, Corcoran RB, Ebi H, Sequist LV, Waltman BA, Chung E, Incio J, Digumarthy SR, Pollack SF, Song Y, Muzikansky A, Lifshits E, Roberge S (2011). BIM expression in treatment-naive cancers predicts responsiveness to kinase inhibitors. Cancer Discov.

[19] Czabotar PE, Lessene G, Strasser A, Adams JM (2014). Control of apoptosis by the BCL-2 protein family: implications for physiology and therapy. Nat Rev Mol Cell Biol.

[20] Faber AC, Ebi H, Costa C, Engelman JA (2012). Apoptosis in targeted therapy responses: the role of BIM. Adv Pharmacol.

[21] Costa C, Molina MA, Drozdowskyj A, Gimenez-Capitan A, Bertran-Alamillo J, Karachaliou N, Gervais R, Massuti B, Wei J, Moran T, Majem M, Felip E, Carcereny E (2014). The impact of EGFR T790M mutations and BIM mRNA expression on outcome in patients with EGFR-mutant NSCLC treated with erlotinib or chemotherapy in the randomized phase III EURTAC trial. Clin Cancer Res.

[22] Ng KP, Hillmer AM, Chuah CT, Juan WC, Ko TK, Teo AS, Ariyaratne PN, Takahashi N, Sawada K, Fei Y, Soh S, Lee WH, Huang JW (2012). A common BIM deletion polymorphism mediates intrinsic resistance and inferior responses to tyrosine kinase inhibitors in cancer. Nat Med.

[23] Nakagawa T, Takeuchi S, Yamada T, Ebi H, Sano T, Nanjo S, Ishikawa D, Sato M, Hasegawa Y, Sekido Y, Yano S (2013). EGFR-TKI resistance due to BIM polymorphism can be circumvented in combination with HDAC inhibition. Cancer Res.

[24] Zheng L, Lin B, Song Z, Xie F, Hong W, Feng J, Shao L, Zhang Y (2013). [Relationship between BIM gene polymorphism and therapeutic efficacy in the retreatment of advanced non-small cell lung cancer with tyrosine kinase inhibitor]. [Article in Chinese]. Zhongguo Fei Ai Za Zhi.

[25] Lee JK, Shin JY, Kim S, Lee S, Park C, Kim JY, Koh Y, Keam B, Min HS, Kim TM, Jeon YK, Kim DW, Chung DH (2013). Primary resistance to epidermal growth factor receptor (EGFR) tyrosine kinase inhibitors (TKIs) in patients with non-small-cell lung cancer harboring TKI-sensitive EGFR mutations: an exploratory study. Ann Oncol.

[26] Zhao M, Zhang Y, Cai W, Li J, Zhou F, Cheng N, Ren R, Zhao C, Li X, Ren S, Zhou C, Hirsch FR (2014). The Bim deletion polymorphism clinical profile and its relation with tyrosine kinase inhibitor resistance in Chinese patients with non-small cell lung cancer. Cancer.

[27] Lee JH, Lin YL, Hsu WH, Chen HY, Chang YC, Yu CJ, Shih JY, Lin CC, Chen KY, Ho CC, Laio WY, Yang PC, Yang JC (2014). Bcl-2-like protein 11 deletion polymorphism predicts survival in advanced non-small-cell lung cancer. J Thorac Oncol.

[28] Sobe K, Hata Y, Tochigi N, Kaburaki K, Kobayashi H, Makino T, Otsuka H, Sato F, Ishida F, Kikuchi N, Hirota N, Sato K, Sano G (2014). Clinical significance of BIM deletion polymorphism in non-small-cell lung cancer with epidermal growth factor receptor mutation. J Thorac Oncol.

[29] Zhong J, Li ZX, Zhao J, Duan JC, Bai H, An TT, Yang XD, Wang J (2014). Analysis of BIM (BCL-2 like 11 gene) deletion polymorphism in Chinese non-small cell lung cancer patients. Thoracic Cancer.

[30] Lee JY, Ku BM, Lim SH, Lee MY, Kim H, Kim M, Kim S, Jung HA, Sun JM, Ahn JS, Park K, Ahn MJ (2015). The BIM Deletion Polymorphism and its Clinical Implication in Patients with EGFR-Mutant Non-Small-Cell Lung Cancer Treated with EGFR Tyrosine Kinase Inhibitors. J Thorac Oncol.

[31] Cardona AFC, Arrieta O, Karachaliou N, Drozdowskyj A, Capitan AG, Molina MA, Martin C, Carranza H, Vargas C, Otero J, Rojas L, Cuello M, Rosell R (2014). BIM deletion polymorphisms in Colombian patients with non-small cell lung cancer who carriers EGFR mutations (clicap). Journal of Thoracic Oncology.

[32] Ying HQ, Chen J, He BS, Pan YQ, Wang F, Deng QW, Sun HL, Liu X, Wang SK (2015). The effect of BIM deletion polymorphism on intrinsic resistance and clinical outcome of cancer patient with kinase inhibitor therapy. Sci Rep.

[33] Nie W, Tao X, Wei H, Chen WS, Li B (2015). The BIM deletion polymorphism is a prognostic biomarker of EGFR-TKIs response in NSCLC: A systematic review and meta-analysis. Oncotarget.

[34] Ma JY, Yan HJ, Gu W (2015). Association between BIM deletion polymorphism and clinical outcome of EGFR-mutated NSCLC patient with EGFR-TKI therapy: A meta-analysis. J Cancer Res Ther.

[35] Huang WF, Liu AH, Zhao HJ, Dong HM, Liu LY, Cai SX (2015). BIM Gene Polymorphism Lowers the Efficacy of EGFR-TKIs in Advanced Nonsmall Cell Lung Cancer With Sensitive EGFR Mutations: A Systematic Review and Meta-Analysis. Medicine (Baltimore).

[36] Riley RD, Lambert PC, Abo-Zaid G (2010). Meta-analysis of individual participant data: rationale, conduct, and reporting. BMJ.

[37] Kim GW, Song JS, Choi CM, Rho JK, Kim SY, Jang SJ, Park YS, Chun SM, Kim WS, Lee JS, Kim SW, Lee DH, Lee JC (2015). Multiple resistant factors in lung cancer with primary resistance to EGFR-TK inhibitors confer poor survival. Lung Cancer.

[38] Li H, Hu H, Wang R, Pan Y, Wang L, Li Y, Zhang Y, Ye T, Li B, Shen L, Sun Y, Chen H (2014). Primary concomitant EGFR T790M mutation predicted worse prognosis in non-small cell lung cancer patients. Onco Targets Ther.

[39] Lim SH, Lee JY, Sun JM, Ahn JS, Park K, Ahn MJ (2014). Comparison of clinical outcomes following gefitinib and erlotinib treatment in non-small-cell lung cancer patients harboring an epidermal growth factor receptor mutation in either exon 19 or 21. J Thorac Oncol.

[40] Kim ST, Uhm JE, Lee J, Sun JM, Sohn I, Kim SW, Jung SH, Park YH, Ahn JS, Park K, Ahn MJ (2012). Randomized phase II study of gefitinib versus erlotinib in patients with advanced non-small cell lung cancer who failed previous chemotherapy. Lung Cancer.

[41] Stewart EL, Tan SZ, Liu G, Tsao MS (2015). Known and putative mechanisms of resistance to EGFR targeted therapies in NSCLC patients with EGFR mutations-a review. Transl Lung Cancer Res.

[42] Galvani E, Peters GJ, Giovannetti E (2012). EGF receptor-targeted therapy in non-small-cell lung cancer: Role of germline polymorphisms in outcome and toxicity. Future Oncology.

[43] Gagne V, Rousseau J, Labuda M, Sharif-Askari B, Brukner I, Laverdiere C, Ceppi F, Sallan SE, Silverman LB, Neuberg D, Kutok JL, Sinnett D, Krajinovic M (2013). Bim polymorphisms: influence on function and response to treatment in children with acute lymphoblastic leukemia. Clin Cancer Res.

[44] Augis V, Airiau K, Josselin M, Turcq B, Mahon FX, Belloc F (2013). A single nucleotide polymorphism in cBIM is associated with a slower achievement of major molecular response in chronic myeloid leukaemia treated with imatinib. PLoS One.

[45] Ishikawa T, Toyoda Y, Yoshiura K, Niikawa N (2012). Pharmacogenetics of human ABC transporter ABCC11: new insights into apocrine gland growth and metabolite secretion. Front Genet.

[46] Panageas KS, Ben-Porat L, Dickler MN, Chapman PB, Schrag D (2007). When you look matters: the effect of assessment schedule on progression-free survival. J Natl Cancer Inst.

[47] Li R, Moudgil T, Ross HJ, Hu HM (2005). Apoptosis of non-small-cell lung cancer cell lines after paclitaxel treatment involves the BH3-only proapoptotic protein Bim. Cell Death Differ.

[48] Savry A, Carre M, Berges R, Rovini A, Pobel I, Chacon C, Braguer D, Bourgarel-Rey V (2013). Bcl-2-enhanced efficacy of microtubule-targeting chemotherapy through Bim overexpression: implications for cancer treatment. Neoplasia.

[49] Atsumi J, Shimizu K, Ohtaki Y, Kaira K, Kakegawa S, Nagashima T, Enokida Y, Nakazawa S, Obayashi K, Takase Y, Kawashima O, Kamiyoshihara M, Sugano M (2015). Impact of the Bim Deletion Polymorphism on Survival Among Patients With Completely Resected Non–Small-Cell Lung Carcinoma. Journal of Global Oncology.

[50] Tanaka A, Sueoka-Aragane N, Nakamura T, Takeda Y, Mitsuoka M, Yamasaki F, Hayashi S, Sueoka E, Kimura S (2012). Co-existence of positive MET FISH status with EGFR mutations signifies poor prognosis in lung adenocarcinoma patients. Lung Cancer.

[51] Yu HA, Arcila ME, Hellmann MD, Kris MG, Ladanyi M, Riely GJ (2014). Poor response to erlotinib in patients with tumors containing baseline EGFR T790M mutations found by routine clinical molecular testing. Ann Oncol.

[52] Arrieta O, Cardona AF, Federico Bramuglia G, Gallo A, Campos-Parra AD, Serrano S, Castro M, Aviles A, Amorin E, Kirchuk R, Cuello M, Borbolla J, Riemersma O (2011). Genotyping non-small cell lung cancer (NSCLC) in Latin America. J Thorac Oncol.

[53] Sterne JA, Egger M, Moher D (2008). Addressing reporting biases. Cochrane handbook for systematic reviews of interventions: Cochrane book series.

