# Supplementary material for: A systematic review and meta-analysis of individual patient data on the impact of the BIM deletion polymorphism on treatment outcomes in epidermal growth factor receptor mutant lung cancer
Source: Oncotarget. 2017 Apr 13;8(25):41474–86. doi: 10.18632/oncotarget.17102 (PMC5522319; doi:10.18632/oncotarget.17102)
Supplement: Supplementary file 1 [file oncotarget-08-41474-s001.pdf]

A systematic review and meta-analysis of individual patient data on the impact of the *BIM* deletion polymorphism on treatment outcomes in epidermal growth factor receptor mutant lung cancer

Supplementary Materials

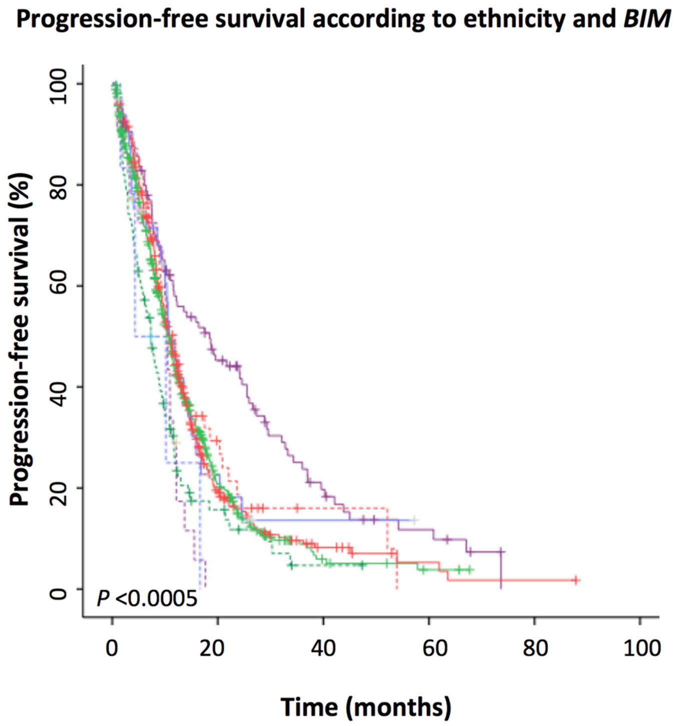

Ethnicity	Genotype	Sample size	PFS	p value
Chinese	With *BIM* deletion	90	7.4	0.004
Chinese	Without *BIM* deletion	534	10.7	
Japanese	With *BIM* deletion	22	10.4	<0.0005
Japanese	Without *BIM* deletion	106	18.4	
Korean	With *BIM* deletion	55	11.9	0.474
Korean	Without *BIM* deletion	354	10.9	
Others	With *BIM* deletion	6	4.3	0.246
Others	Without *BIM* deletion	33	10.7	

Supplementary Figure 1: Kaplan-Meier curves comparing progression-free survival (PFS) stratified according to ethnicity as indicated by the colour coding shown. PFS curves of patients with the *BIM* deletion are shown as dotted lines.

Supplementary Table 1: Number of patients used from each study and reason for exclusion

Author	Year	Country	Patients contributed/ used/excluded	Reason for exclusion of patients
Ng KP *et al.*	2012	SingaporeJapan	141/137/4	4 patients treated for ≤ 0.5 months
Lee JK *et al.*	2013	South Korea	197/191/6	4 patients had unknown *BIM* genotype, 2 patients treated for ≤ 0.5 months
Isobe K *et al.*	2014	Japan	70/67/3	3 patients treated for ≤ 0.5 months
Lee JH *et al.*	2014	Taiwan	153/152/1	1 patient treated for ≤ 0.5 months
Zhao MC *et al.*	2014	China	166/164/2	2 patients treated for ≤ 0.5 months
Kim GW *et al.*	2015	South Korea	18/18/0	N.A.
Lee JY *et al.*	2015	South Korea	205/200/5	3 patients treated for ≤ 0.5 months
Yano S *et al.*	Unpublished	Japan	39/38/1	1 patient treated for ≤ 0.5 months
Lim WT *et al.*	Unpublished	Singapore	245/178/67	59 patients duplicated in Ng *et al.*, 1 person lost to followup after diagnosis, 7 patients treated for <0.5 months
Lu S *et al.*	Unpublished	China	90/55/35	35 patients were not treated with EGFR-TKIs
		**Total**	1324/1200/124	

Supplementary Table 2: Clinical characteristics of patients with and without the *BIM* deletion

		Entire cohort (*n* = 1200)		Without *BIM* deletion (*n* = 1027)		With *BIM* deletion (*n* = 173)		*p* value
**Age**	Mean age at diagnosis	60.5		60.4		60.7		0.787
**Sex**	Male (% of total)	457	(38.1%)	393	(38.3%)	64	(37.0%)	0.800
	Female (% of total)	743	(61.9%)	634	(61.7%)	109	(63.0%)	
**Ethnicity**	Chinese	624	(52.0%)	534	(52.0%)	90	(52.0%)	0.768
	Japanese	128	(10.7%)	106	(10.3%)	22	(12.7%)	
	Korean	409	(34.1%)	354	(34.5%)	55	(31.8%)	
	Other	39	(3.2%)	33	(3.2%)	6	(3.5%)	
**ECOG status**	0–1	1071	(89.2%)	916	(89.2%)	155	(89.6%)	0.643
	≥ 2	88	(7.3%)	74	(7.2%)	14	(8.1%)	
	Unknown	41	(3.4%)	37	(3.6%)	4	(2.3%)	
**Smoking history**	Never smoker	306	(25.5%)	263	(25.6%)	43	(24.9%)	0.824
	Ex/current smoker	892	(74.3%)	762	(74.2%)	130	(75.1%)	
	Unknown	2	(0.2%)	2	(0.2%)	0	(0.0%)	
**Disease stage**	IIIA/IIIB	102	(8.5%)	89	(8.7%)	13	(7.5%)	0.881
	IV	914	(76.2%)	781	(76.0%)	133	(76.9%)	
	Postoperative relapse	184	(15.3%)	157	(15.3%)	27	(15.6%)	
**Histology**	Adenocarcinoma	1134	(94.5%)	967	(94.2%)	167	(96.5%)	0.278
	Non-adenocarcinoma	66	(5.5%)	60	(5.8%)	6	(3.5%)	
***EGFR* exon mutated**	18	19	(1.6%)	17	(1.7%)	2	(1.2%)	0.255
	19	628	(52.3%)	536	(52.2%)	92	(53.2%)	
	20	8	(0.7%)	6	(0.6%)	2	(1.2%)	
	21	431	(35.9%)	371	(36.1%)	60	(34.7%)	
	Complex	29	(2.4%)	24	(2.3%)	5	(2.9%)	
	Wildtype	49	(4.1%)	38	(3.7%)	11	(6.4%)	
	Not tested	36	(3.0%)	35	(3.4%)	1	(0.6%)	
**TKI used**	Gefitinib	867	(72.2%)	735	(71.6%)	132	(76.3%)	0.389
	Erlotinib	291	(24.2%)	253	(24.6%)	38	(22.0%)	
	Geftinib and Erlotinib	14	(1.2%)	14	(1.4%)	0	(0.0%)	
	Icotinib or Afatinib	28	(2.4%)	25	(2.4%)	3	(1.7%)	
**Line of treatment**	First-line	687	(57.2%)	584	(56.9%)	103	(59.5%)	0.653
	Later	509	(42.2%)	440	(42.8%)	69	(39.9%)	
	Unknown	4	(0.3%)	3	(0.3%)	1	(0.6%)	

Supplementary Table 3: Clinical characteristics of patients of Korean and non-Korean ethnicity

		Entire cohort (*n* = 1200)	Non-Korean (*n* = 791)	Korean (*n* = 409)	*p* value
**Age**	Mean age at diagnosis	60.5	60.6	60.1	0.441
**Sex**	Male (% of total)	457 (38.1%)	317 (40.1%)	140 (34.2%)	0.052
	Female (% of total)	743 (61.9%)	474 (59.9%)	269 (65.8%)	
**ECOG status**	0–1	1071 (89.2%)	718 (90.8%)	353 (86.3%)	0.041
	≥ 2	88 (7.3%)	52 (6.6%)	36 (8.8%)	
	Unknown	41 (3.4%)	21 (2.6%)	20 (4.9%)	
**Smoking history**	Never smoker	306 (25.5%)	590 (74.6%)	302 (73.8%)	0.560
	Ex/current smoker	892 (74.3%)	199 (25.2%)	107 (26.2%)	
	Unknown	2 (0.2%)	2 (0.2%)	0 (0.0%)	
**Disease stage**	IIIA/IIIB	102 (8.5%)	75 (9.5%)	27 (6.6%)	0.101
	IV/Postoperative relapse	1098 (91.5%)	716 (90.5%)	382 (93.4%)	
**Histology**	Adenocarcinoma	1134 (94.5%)	733 (92.7%)	401 (98.0%)	< 0.0005
	Non-adenocarcinoma	66 (5.5%)	58 (7.3%)	8 (0.2%)	
***EGFR* exon mutated**	18	19 (1.6%)	12 (1.5%)	7 (1.7%)	< 0.0005
	19	628 (52.3%)	393 (49.7%)	235 (57.5%)	
	20	8 (0.7%)	8 (1.0%)	0 (0.0%)	
	21	431 (35.9%)	273 (34.5%)	158 (38.6%)	
	Complex	29 (2.4%)	20 (2.5%)	9 (2.2%)	
	Wildtype	49 (4.1%)	49 (6.2%)	0 (0.0%)	
	Not tested	36 (3.0%)	36 (4.6%)	0 (0.0%)	
**TKI used**	Gefitinib	867 (72.2%)	553 (69.9%)	314 (76.8%)	< 0.0005
	Erlotinib	291 (24.2%)	196 (24.8%)	95 (23.2%)	
	Geftinib and Erlotinib	14 (1.2%)	14 (1.8%)	0 (0.0%)	
	Icotinib or Afatinib	28 (2.4%)	28 (3.5%)	0 (0.0%)	
**Line of treatment**	First-line	687 (57.2%)	540 (68.3%)	147 (35.9%)	< 0.0005
	Later	509 (42.2%)	247 (31.2%)	262 (64.1%)	
	Unknown	4 (0.3%)	4 (0.5%)	0 (0.0%)	

Supplementary Table 4: Subgroup analysis, non-Korean *v* Korean

	PFS (months) in non-Koreans		*p* value	PFS (months) in Koreans		*p* value
**Subgroup**	**Without *BIM* deletion**	**With *BIM* deletion**		**Without BIM deletion**	**With *BIM* deletion**	
ECOG of 0 or 1	11.3	8.9	< 0.0005	10.8	10.7	0.311
Adenocarcinoma histology	11.3	8.0	< 0.0005	11.0	11.9	0.525
Sensitizing *EGFR* mutation	11.7	9.4	< 0.0005	10.9	11.9	0.391
Gefitinib and/or erlotinib	11.1	7.9	< 0.0005	10.9	11.9	0.474
First-line treatment	10.1	8.3	0.004	9.9	10.5	0.803

Supplementary Table 5: Risk of bias assessment

	Bias due to confounding	Bias in selection of participants into the study	Bias in measurement of interventions	Bias due to departure from intended intervention	Bias due to missing data	Bias in measurement of outcomes	Bias in selection of reported result	Overall risk of bias
**Study**								
Ng KP *et al.*	Low	Low	Low	Low	Low	Low	Low	Low
Lee JK *et al.*	Low	Low	Low	Low	Low	Low	Low	Low
Zheng L *et al.*	Low	Low	Low	Low	Low	Low	Low	Low
Isobe K *et al.*	Low	Low	Low	Low	Low	Low	Low	Low
Lee JH *et al.*	Low	Low	Low	Low	Low	Low	Low	Low
Zhao MC *et al.*	Low	Low	Low	Low	Low	Low	Low	Low
Zhong J *et al.*	Low	Low	Low	Low	Low	Low	Low	Low
Cardona AF *et al.*	Low	Low	Low	Low	Low	Low	Low	Low
Kim GW *et al.*	Low	Low	Low	Low	Low	Low	Low	Low
Lee JY *et al.*	Low	Low	Low	Low	Low	Low	Low	Low
Yano S *et al.*	Low	Low	Low	Low	Low	Low	Low	Low
Lu S *et al.*	Low	Low	Low	Low	Low	Low	Low	Low
Lim WT *et al.*	Low	Low	Low	Low	Low	Low	Low	Low
**Comments**		With the exception of Lee JH *et al.*, all studies were retrospectives studies that genotyped groups of patients for which DNA was available, after treatment outcomes had been measured.	DNA from peripheral blood or FFPE was used to determine *BIM* genotype according to published protocols.	The *BIM* deletion is a germline mutation.	Only patients with known *BIM* genotype were used.	RECIST criteria were used by all centers to determine progression		

Supplementary Table 6: Calculated HRs for *BIM* deletion on PFS for S Lu *et al*

	Univariate Analysis		Multivariate Analysis	
	HR (95% CI)	*p* value	HR (95% CI)	*p* value
**Age at diagnosis**	1.005 (0.97–1.041)	0.786	-	-
**Gender (Ref = Female)**				
Male	1.267 (0.664–2.421)	0.474	-	-
**Smoking history (Ref = Never smoker)**				
Ever smoker	1.088 (0.525–2.254)	0.820	-	-
**Stage (Ref = Relapse)**				
IIIA/B	2.161 (0.658–7.100)	0.204	5.185 (1.068–25.171)	0.041
IV	2.542 (0.972–6.644)	0.057	6.756 (1.657–27.539)	
**Histology (Ref = Adenocarcinoma)**				
Non-adenocarcinoma	4.505 (1.515–13.514)	0.007	13.889 (2.762–20.014)	0.001
**EGFR phenotype (Ref = Sensitizing)**				
Resistant	3.614 (1.558–8.379)	0.003	3.220 (1.339–7.744)	0.009
**Line of treatment (Ref = Later)**				
First-line	1.528 (0.780–2.993)	0.216	–	-
**BIM deletion (Ref = Absent)**				
Present	1.425 (0.651–3.12)	0.376	1.616 (0.699–3.738)	0.262

Supplementary Table 7: Calculated HRs for *BIM* deletion on PFS for S Yano *et al*

	Univariate Analysis		Multivariate Analysis	
	**HR (95% CI)**	***p* value**	**HR (95% CI)**	***p* value**
**Age at diagnosis**	0.985 (0.956–1.015)	0.314	-	-
**Gender (Ref = Female)**				
Male	1.912 (0.912–4.000)	0.086	2.227 (1.024–4.854)	0.043
**Smoking history (Ref = Never smoker)**				
Ever smoker	1.250 (0.601–2.602)	0.551	–	-
**Stage (Ref = Relapse)**				
IIIA/B	0.669 (0.176–2.548)	0.556	0.565 (0.148–2.160)	0.404
IV	0.473 (0.17–1.313)	0.151	0.410 (0.141–1.218)	0.109
**EGFR phenotype (Ref = Sensitizing)**				
Resistant	1.076 (0.322–3.594)	0.906	–	-
**Line of treatment (Ref = Later)**				
First-line	0.873 (0.415–1.839)	0.721	–	-
**ECOG status (Ref = 0 or 1)**				
≥ 2	1.525 (0.205–11.319)	0.680	–	-
**BIM deletion (Ref = Absent)**				
Present	0.884 (0.302–2.588)	0.823	0.758 (0.235–2.446)	0.643

Supplementary Table 8: Calculated HRs for *BIM* deletion on PFS for W-T Lim *et al*

	Univariate Analysis		Multivariate Analysis	
	**HR (95% CI)**	***p* value**	**HR (95% CI)**	***p* value**
**Age at diagnosis**	0.992 (0.973–1.011)	0.411	-	-
**Gender (Ref = Female)**				
Male	1.240 (0.835–1.845)	0.285	-	–
**Smoking history (Ref = Never smoker)**				
Ever smoker	0.801 (0.489–1.312)	0.378	-	-
**Stage (Ref = Relapse)**				
IIIA/B	1.750 (0.380–8.051)	0.473	-	
IV	2.203 (0.538–9.021)	0.272	-	-
**Histology (Ref = Adenocarcinoma)**				
Non-adenocarcinoma	2.558 (1.116–5.882)	0.026	2.525 (1.098–2.525)	0.029
**EGFR phenotype (Ref = Sensitizing)**				
Resistant	1.451 (0.458–4.592)	0.527	-	-
**Line of treatment (Ref = Later)**				
First-line	0.890 (0.513–1.544)	0.678	-	-
**ECOG status (Ref = 0 or 1)**				
≥ 2	1.515 (0.697–3.289)	0.294	-	-
**BIM deletion (Ref = Absent)**				
Present	0.880 (0.491–1.577)	0.667	0.918 (0.510–1.650)	0.774

Supplementary Table 9: Calculated HRs for *BIM* deletion on PFS for JK Lee *et al*

	Univariate Analysis			Multivariate Analysis		
	HR (95% CI)		*p* value	HR (95% CI)		*p* value
**Age at diagnosis**	0.985	(0.970–1.000)	0.051	0.984	(0.969–1)	0.048
**Gender (Ref = Female)**						
Male	1.230	(0.872–1.736)	0.238	–		–
**Smoking history (Ref = Never smoker)**						
Ever smoker	1.112	(0.764–1.618)	0.579	–		–
**Stage (Ref = Relapse)**						
IIIA/B	1.311	(0.717–2.397)	0.379	1.265	(0.683–2.344)	0.455
IV	0.748	(0.504–1.109)	0.149	0.790	(0.528–1.180)	0.249
**Histology (Ref = Adenocarcinoma)**						
Non-adenocarcinoma	2.101	(0.855–5.155)	0.105	0.466	(0.184–1.184)	0.109
**EGFR phenotype (Ref = Sensitizing)**						
Resistant	1.675	(0.413–6.79)	0.470	–		–
**Line of treatment (Ref = Later)**						
First-line	0.917	(0.638–1.317)	0.638	–		–
**ECOG status (Ref = 0 or 1)**						
≥ 2	0.801	(0.442–1.453)	0.466	–		–
**BIM deletion (Ref = Absent)**						
Present	1.038	(0.596–1.806)	0.896	1.022	(0.579–1.803)	0.941

Supplementary Table 10: Calculated HRs for *BIM* deletion on PFS for JY Lee *et al*

	Univariate Analysis			Multivariate Analysis		
	HR (95% CI)		*p* value	HR (95% CI)		*p* value
**Age at diagnosis**	0.988	(0.973–1.003)	0.122	0.987	(0.972–1.002)	0.095
**Gender (Ref = Female)**						
Male	1.623	(1.166–2.262)	0.004	0.711	(0.432–1.171)	0.180
**Smoking history (Ref = Never smoker)**						
Ever smoker	1.607	(1.115–2.315)	0.011	1.130	(0.651–1.964)	0.663
**Stage (Ref = Relapse)**						
IIIA/B	2.193	(1.034–4.654)	0.041	2.139	(0.987–4.637)	0.054
IV	1.491	(1.074–2.071)	0.017	1.520	(1.084–2.132)	0.015
**Histology (Ref = Adenocarcinoma)**						
Non-adenocarcinoma	0.207	(0.05–0.856)	0.030	0.204	(0.047–0.89)	0.034
**Line of treatment (Ref = Later)**						
First-line	1.020	(0.735–1.414)	0.908	–		–
**ECOG status (Ref = 0 or 1)**						
≥ 2	1.692	(0.931–3.067)	0.084	0.563	(0.309–1.026)	0.061
**BIM deletion (Ref = Absent)**						
Present	0.799	(0.529–1.206)	0.285	0.846	(0.558–1.283)	0.431

Supplementary Table 11: Calculated HRs for *BIM* deletion on PFS for GW Kim *et al*

	Univariate Analysis		Multivariate Analysis		
	HR (95% CI)	*p* value	HR (95% CI)		*p* value
**Age at diagnosis**	1.037 (0.994–1.081)	0.090	1.164	(1.057–1.282)	0.002
**Gender (Ref = Female)**					
Male	1.736 (0.641–4.695)	0.278	–		–
**Smoking history (Ref = Never smoker)**					
Ever smoker	1.353 (0.487–3.761)	0.562	–		–
**Stage (Ref = IV)**					
IIIA/B	0.953 (0.122–7.443)	0.963	–		–
**Line of treatment (Ref = Later)**					
First-line	0.324 (0.094–1.117)	0.074	0.069	(0.011–0.419)	0.004
**ECOG status (Ref = 0 or 1)**					
≥ 2	0.125 (0.011–1.382)	0.090	0.033	(0.002–0.58)	0.020
**BIM deletion (Ref = Absent)**					
Present	0.309 (0.067–1.434)	0.134	0.034	(0.003–0.442)	0.010
